# Ghrelin octanoylation by ghrelin *O*-acyltransferase: protein acylation impacting metabolic and neuroendocrine signalling

**DOI:** 10.1098/rsob.210080

**Published:** 2021-07-28

**Authors:** Tasha R. Davis, Mariah R. Pierce, Sadie X. Novak, James L. Hougland

**Affiliations:** ^1^ Department of Chemistry, Syracuse University, Syracuse, NY 13244 USA; ^2^ BioInspired Syracuse, Syracuse University, Syracuse, NY 13244 USA

**Keywords:** ghrelin, ghrelin *O*-acyltransferase, GHS-R1a, membrane-bound *O*-acyltransferase, neuroendocrinology, protein acylation

## Abstract

The acylated peptide hormone ghrelin impacts a wide range of physiological processes but is most well known for controlling hunger and metabolic regulation. Ghrelin requires a unique posttranslational modification, serine octanoylation, to bind and activate signalling through its cognate GHS-R1a receptor. Ghrelin acylation is catalysed by ghrelin *O*-acyltransferase (GOAT), a member of the membrane-bound *O*-acyltransferase (MBOAT) enzyme family. The ghrelin/GOAT/GHS-R1a system is defined by multiple unique aspects within both protein biochemistry and endocrinology. Ghrelin serves as the only substrate for GOAT within the human proteome and, among the multiple hormones involved in energy homeostasis and metabolism such as insulin and leptin, acts as the only known hormone in circulation that directly stimulates appetite and hunger signalling. Advances in GOAT enzymology, structural modelling and inhibitor development have revolutionized our understanding of this enzyme and offered new tools for investigating ghrelin signalling at the molecular and organismal levels. In this review, we briefly summarize the current state of knowledge regarding ghrelin signalling and ghrelin/GOAT enzymology, discuss the GOAT structural model in the context of recently reported MBOAT enzyme superfamily member structures, and highlight the growing complement of GOAT inhibitors that offer options for both ghrelin signalling studies and therapeutic applications.

## Graphical abstract

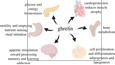

## 1. Introduction: the discovery of ghrelin octanoylation and ghrelin *O*-acyltransferase

Ghrelin was discovered in 1999 by Kojima *et al*. [1] while searching for the endogenous ligand for the orphan growth hormone secretagogue receptor GHS-R1a. To identify the unknown ligand for GHS-R1a, CHO cells expressing rat GHS-R1a were used to screen a range of rat tissue extracts for receptor activation. Using the extract from rat stomach tissue, ghrelin was purified and characterized via Edman degradation as a 28 amino acid peptide. Curiously, the sequence of all 28 amino acids was determined except for the third residue in this initial characterization. Functional comparison of chemically synthesized ghrelin to purified ghrelin showed the synthetic peptide did not activate the GHS-R1a receptor as indicated by an increase in cellular Ca^2+^ levels in GHS-R1a-expressing cells. This lack of signalling activity with the synthetic ghrelin peptide indicated ghrelin may contain an unanticipated chemical modification required for biological activity.

Based on the failure of Edman degradation to identify the serine residue at the third position (Ser-3) and the lack of biological activity exhibited by synthetic ghrelin, Kojima and co-workers hypothesized that this residue undergoes a functionally essential posttranslational modification to produce active ghrelin. Reverse-phase HPLC analysis indicated purified ghrelin exhibited markedly higher hydrophobic character than synthetic ghrelin, and mass spectrometry determined a 126 Da mass difference between purified ghrelin (3315 Da) and synthetic ghrelin (3189 Da). These findings led to the conclusion that the Ser-3 residue was acylated with n-octanoic acid, a previously unknown protein posttranslational modification [1]. Synthetic ghrelin was produced with Ser-3 acylated with n-octanoic acid, and this octanoylated synthetic ghrelin matched purified ghrelin in RP-HPLC and mass spectrometry characterization. Furthermore, the octanoylated synthetic peptide increased calcium concentrations in cells expressing GHS-R1a consistent with receptor activation [1]. Since the discovery of ghrelin and its octanoyl serine residue, no other examples of this posttranslational modification have been reported in other proteins [2–4].

Nearly a decade after the discovery of ghrelin and its unique octanoylated serine modification, the enzyme that catalyses ghrelin acylation was identified in 2008 by two independent research groups as MBOAT4, a member of the membrane-bound *O*-acyltransferase (MBOAT) superfamily [3,5]. The MBOAT enzyme superfamily was first described in 2000, and proteins belonging to this family are characterized by multiple transmembrane domains and two conserved functionally essential residues including an invariant histidine [6,7]. The studies identifying MBOAT4, subsequently renamed ghrelin *O*-acyltransferase (GOAT), as the enzyme responsible for acylating ghrelin acylating enzyme used complementary approaches. Yang and co-workers used MBOAT candidate overexpression to demonstrate ghrelin acylation activity by GOAT while Gutierrez and co-workers employed MBOAT gene silencing to determine which (if any) MBOAT member was required to mediate ghrelin acylation [3,5]. Silencing of MBOAT4 alone resulted in a decrease of ghrelin acylation with subsequent transfection with MBOAT4 reestablishing ghrelin acylation [5]. Through these landmark studies, GOAT was added as the third essential protein involved in the ghrelin signalling pathway alongside ghrelin and GHS-R1a.

## 2. Role of acylation in ghrelin signalling

### 2.1. A brief overview of physiological processes impacted by ghrelin signalling

**Figure 1.**
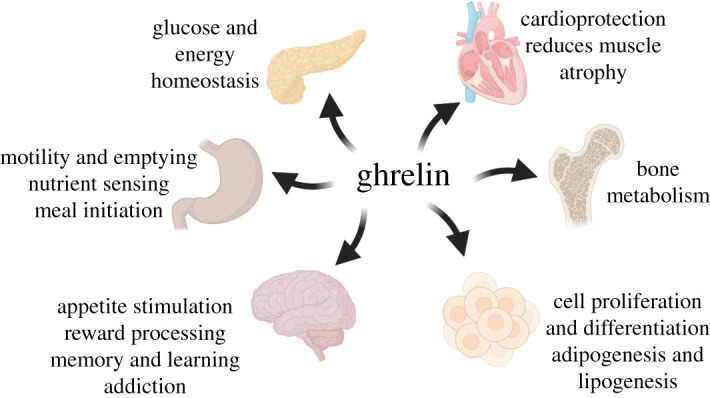
Ghrelin signalling impacts a wide range of physiological pathways. Ghrelin is involved in a multitude of pathways including appetite stimulation, glucose homeostasis, adipogenesis, cardiovascular health and modulation of addictive behaviour. Figure created with BioRender.com.

Ghrelin was first observed to be responsible for the regulation of nutrient sensing, meal initiation and appetite, giving ghrelin its most well-known nickname as the ‘hunger hormone’ [8,9]. However, ghrelin exerts its influence on multiple physiological pathways beyond its original linkage to growth hormone secretion and appetite regulation (figure 1) [9]. These pathways include glucose metabolism and energy homeostasis [10–15], organismal response to starvation [16–19], cardio-protection [20,21], protection against muscle atrophy [22–24] and bone metabolism [9,25]. Recent studies suggest ghrelin is involved in the mediation of behavioural and biochemical effects of drugs linked to addiction and addictive behaviour [26–31]. Ghrelin is also linked to signalling pathways linked to learning, memory, reward processing and stress response [32–38]. Due to this wide range of physiological signalling functions, the ghrelin signalling pathway and its associated proteins are considered attractive unexploited targets for therapeutic development and clinical application [39].

### 2.2. Recognition of ghrelin by GHS-R1a

At the time of its discovery, it was established that ghrelin must undergo a serine acylation modification to become competent to activate signalling through its cognate receptor. Once acylated, acyl ghrelin (hereafter referred to as ‘ghrelin’) can bind to its receptor, the growth hormone secretagogue receptor (GHS-R). GHS-R is a rhodopsin-like heterotrimeric G-coupled protein of β-branch in the class A GPCRs [40–44]. The receptor has two identified expressed isoforms, GHS-R1a and GHS-R1b [42]. Human GHS-R1a comprises 377 amino acids in seven transmembrane helices and is the functional receptor that binds ghrelin [42,43,45]. The receptor contains three conserved residues Glu140-Arg141-Tyr142 located at the intracellular end of transmembrane helix 3 (TM 3) which are critical for the isomerization of the receptor between its active and inactive conformations. The GHS-R1b is a truncated splice variant lacking TM 6 and TM 7, and is not able to bind ghrelin or transduce ghrelin signalling [46,47]. While GHS-R1b does not itself exhibit biological signalling, it is suggested to act as a modulator of GHS-R1a signalling through impact on GHS-R1a cellular trafficking and formation of GHS-R1a signalling complexes with other receptors [42,44,47,48]. Both variants are expressed in brain regions including the hypothalamus, hippocampus, amygdala, mesencephalic dopaminergic regions and striatum, with GHS-R1a recognized as the functional ghrelin receptor [47,49].

**Figure 2.**
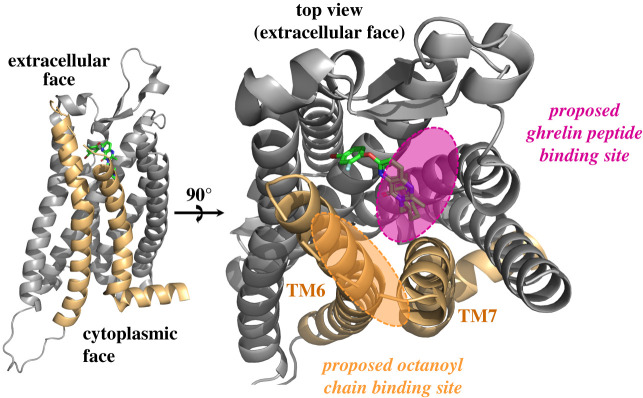
GHS-R1a structure reveals a proposed binding for ghrelin. The binding site for ghrelin is composed of two regions on the receptor extracellular face, with polar contact to amino acids for contacts with the ghrelin N-terminal amino acids and a nonpolar surface for interaction with the octanoyl group attached through the serine ester. Figure created in PyMol using PDB 6KO5.

Like many GPCRs, GHS-R1a exhibits both ligand-dependent and ligand-independent intracellular signalling [39,45,50]. While this latter constitutive signalling is commonly observed at low levels compared to ligand-activated signalling, GHS-R1a ligand-independent signalling activity was found to be the highest of any known constitutive GPCR at about 50% its capacity when its ligand is bound [50]. While the structural basis of both constitutive and acylated ghrelin-dependent ligand-activated GHS-R1a signalling have yet to be fully defined, structural analyses of the receptor have provided insight into potential mechanisms [44]. Investigating the mechanism of the GHS-R1a constitutive activity, Holst *et al*. revealed a key cluster of aromatic residues between transmembrane helices (TM) 6 and 7 (Phe-16, TM6; Phe-6, TM7; Phe-9, TM7) [45,50]. It was suggested the proximity of these residues allowed for interactions that stabilize the receptor in its active form in the absence of a bound agonist [45]. In a recent study reporting the structure of GHS-R1a bound to an antagonist, Shiimura *et al*. [44] found the ligand-binding pocket of GHS-R1a is bifurcated by a salt bridge formed between Arg-283 (TM6) and Glu-124 (TM3). This feature is unique to GHS-R1a compared to other structurally characterized peptide hormone GPCRs. One side of the cavity is rich in polar residues which are important for ligand binding in other peptide hormone GPCRs (figure 2). Mutating polar residues to alanine within this region causes a significant loss of receptor activity [44]. This structure also showed a wide gap between TM6 and TM7 rich with hydrophobic residues, with a cluster of phenylalanine residues between these helices that are essential for ligand binding. GHS-R1a also contains two conserved cysteine residues (Cys-116 and Cys-198) which form a disulfide bond, with disruption of this disulfide completely abolishing receptor function [51].

### 2.3. Biological signalling by acyl ghrelin

Upon ghrelin binding to GHS-R1a, *α* and *βγ* subunits of heterotrimeric G proteins are disassociated, activating downstream signal pathways through a series of interactions with various intracellular molecules [43,45]. Several signalling pathways linked to GHS-R1a result in an increase in intracellular Ca^2+^ concentration. Through the G*α*_q_ coupled pathway, downstream signalling includes triggering inositol phosphate production, resulting in the mobilization of intracellular calcium ions and ultimately the release of growth hormone (GH) [43,45,48,52]. Another signalling pathway cascade resulting in the increase of Ca^2+^ ions involves hypothalamic ghrelin activation of agouti-related peptide/neuropeptide Y-containing neurons (AgRP/NYP) and the inhibition of pro-opiomelanocortin (POMC) neurons in the arcuate nucleus of the hypothalamus [43,48,53]. The change in Ca^2+^ levels is dependent on the influx of Ca^2+^ ions through the N-type calcium channel, which is activated by the cAMP/PKA signalling pathway and upon the coupling of GHS-R1a to a G*α*_s_ protein [43,54].

GHS-R1a is also involved AMP-activated protein kinase (AMPK) signalling. AMPK is proposed to mediate ghrelin's effect on energy homeostasis and metabolism, eliciting tissue-dependent effects [43,55]. Ghrelin also modulates the phosphoinositide 3-kinase/protein kinase B (PI3 K/AKT) signalling pathways upon GHS-R1a activation of the insulin receptor substrate (IRS-1) [43,56]. The PI3 K/AKT has diverse downstream effects, including glucose metabolism, biosynthesis of macromolecules, maintenance of redox balances and plays an essential role for cell survival and growth [56]. Similar to other ghrelin-dependent signalling pathways, the downstream effects of ghrelin signalling are tissue dependent [43]. In hepatoma cells, exogenous ghrelin stimulates the upregulation of several activities naturally induced by insulin [57]. These activities include tyrosine phosphorylation of IRS-1, an association of the adapter molecule growth factor receptor-bound protein 2 with IRS-1, and cell proliferation [43,57]. In these cells, AKT kinase activity was inhibited and gluconeogenesis was upregulated by reversing the downregulation effects caused by increased insulin levels [43,57]. Though the molecular mechanisms are unknown, cardio-protection effects resulting from ghrelin stimulation is proposed to involve the GHS-R1a mediated activation of the PI3 K/AKT and MAPK signalling pathways [43,58,59].

Ghrelin is also hypothesized to modulate the signalling pathways associated with the mechanistic target of rapamycin (mTOR) [43]. The mTOR pathway is a key nutrient-sensitive regulator for a range of physiological, metabolic and ageing processes in animals [60]. The molecular link between ghrelin and the mTOR pathway has yet to be determined, exogenous ghrelin upregulates the hypothalamic mTOR signalling pathway [53]. In a study by Martins *et al*., upon administering exogenous ghrelin into the brain or the periphery, feeding was stimulated, which was not observed with other orexigenic hormones [53,54]. When the mTOR signalling pathway is inhibited, the orexigenic effects of ghrelin are notably decreased and the mRNA expression of AgRP and NPY was normalized as well as their downstream transcription factors [53].

Other signalling pathways involving ghrelin are associated with cellular proliferation and differentiation through the mitogen-activated protein kinase (MAPK) signalling pathway in several cell types [43,61]. In a study by Delhanty *et al*., the extracellular-signal-regulated kinase (ERK) pathway as well as the PI3 K pathway was inhibited. Upon inhibition of either pathway, the proliferative response of the osteoblasts in the presence of ghrelin was abolished or suppressed [61]. Reflective in the results in this study, multiple signalling pathways may be intersecting with MAPK activation. In another study, ghrelin-mediated activation of MAPK had a negative impact on the survival of immortalized rat podocytes [62]. In other pathways, ghrelin stimulation activates ERK1/2 through β-arrestin-dependent and independent pathways, as well as PI3 K/AKT to promote apoptosis of preosteoblastic and preadipocytic cells [63].

### 2.4. Biological signalling by des-acyl ghrelin

To bind and activate GHS-R1a, ghrelin must be acylated [64]. As a result, the unacylated form of ghrelin (des-acyl ghrelin) was thought to be a nonfunctional peptide [65–67]. However, des-acyl ghrelin has been shown to modulate a range of physiological processes, both independently of and in opposition to acyl ghrelin [68,69]. For example, treatment with either des-acyl ghrelin or ghrelin can stimulate insulin release in β-cell lines [70,71]. Treatment with des-acyl ghrelin promotes cell survival in some β-cell lines [70,71], and des-acyl ghrelin was found to stimulate cell proliferation and inhibit apoptosis induced by serum starvation and cytokine activation in pancreatic β-cells [70]. Des-acyl ghrelin also increased insulin sensitivity in liver, muscle, and adipose tissues and suppressed genes associated with lipid metabolism and lipogenesis in these tissues [68]. The molecule is also proposed to play a role in the modulation of cellular and body growth through its ability to promote the proliferation of human osteoblasts [61]. In rodent bone marrow cells, it has been determined that des-acyl ghrelin promotes adipogenesis [67]. However, expression of des-acyl ghrelin has also been observed to suppress adipogenesis and fat accumulation in other studies suggesting this behaviour may be context-dependent [72]. In glucose-intolerant mice, des-acyl ghrelin provides a protective effect on skeletal muscle and endothelial cells that are subjected to ischaemia conditions [73]. Des-acyl ghrelin treatment also led to the prevention of cellular damage through activation of endothelial oxidative defense [69]. In another study, incubation of human differentiating omental adipocytes with des-acyl ghrelin impeded TNF-α-induced activation of caspase-8 and caspase-3 leading to a reduction in apoptosis. Des-acyl ghrelin reduced TNF-α-induced expression of ATG5, BECN1 and ATG7 genes, which are associated with autophagy [74].

With des-acyl ghrelin incapable of binding and activating the GHS-R1a receptor, the mechanism for des-acyl ghrelin signalling remains undefined. However, several possibilities have been proposed. One potential signalling route would be through a novel des-acyl receptor for which no candidates have yet been identified. Des-acyl ghrelin could also impact ghrelin signalling through the modulation of ‘ghrelin buffering’ by binding partners in circulation. Both ghrelin and des-acyl ghrelin interact with a number of biomolecules in circulation, including lipoproteins, such as very low-density lipoprotein (VLDL), low-density lipoprotein (LDL) and high-density lipoprotein (HDL) [75–77]. The binding of des-acyl ghrelin and ghrelin to HDL suggests that the formation of the HDL-ghrelin complex could serve as a ghrelin reservoir for modulating the levels of acyl ghrelin in circulation [78]. Finally, a recently proposed model for des-acyl ghrelin signalling involves ghrelin re-acylation at the site of cellular signalling [49,67,79]. This mechanism, discussed further later in this review, would require GOAT localization to the cell surface.

## 3. Comparison of MBOAT substrate processing: ghrelin/GOAT, Wnt/PORCN and Hedgehog/Hhat

**Figure 3.**
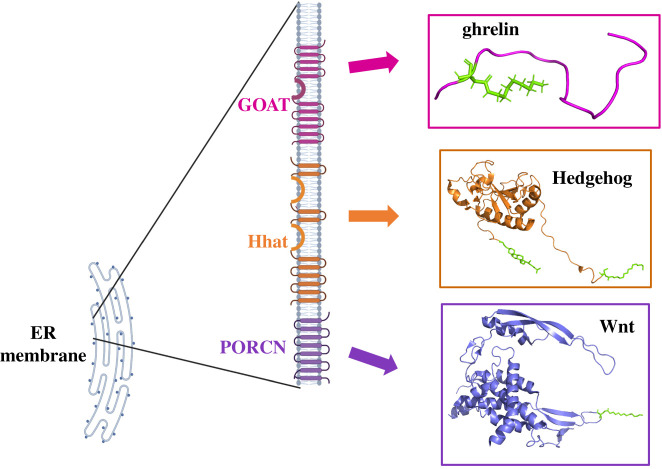
MBOAT family acyltransferases with protein substrates. GOAT, Hhat and PORCN catalyse acylation of their respective substrates (ghrelin, Hedgehog and Wnt) as these proteins proceed through the ER on the secretion pathway. Figure created with BioRender.com and PyMOL using PDB files 6RVD (Hedgehog), 6H3E (ghrelin) and 4FOA (Wnt).

GOAT, Hedgehog acyltransferase (Hhat) and Porcupine (PORCN) constitute a subgroup of acyltransferases in the MBOAT family that modify secreted peptide/protein substrates that play key roles in intercellular signalling (figure 3) [7,80]. Their substrates include the appetite-stimulating peptide ghrelin and the morphogens hedgehog (Hh) and Wingless (Wnt). These acylated proteins play essential roles in physiological energy regulation, cell development, and tissue growth and homeostasis. Ghrelin, Hh and Wnt are modified in the ER lumen where they are acylated by an MBOAT member using fatty acyl-CoA drawn from the cytoplasm to act as an acyl donor [7,81–84]. We briefly compare the expression, processing and modification of these three acylated proteins, with other articles in this issue addressing Hhat/Hedgehog and PORCN/Wnt in more detail.

**Figure 4.**
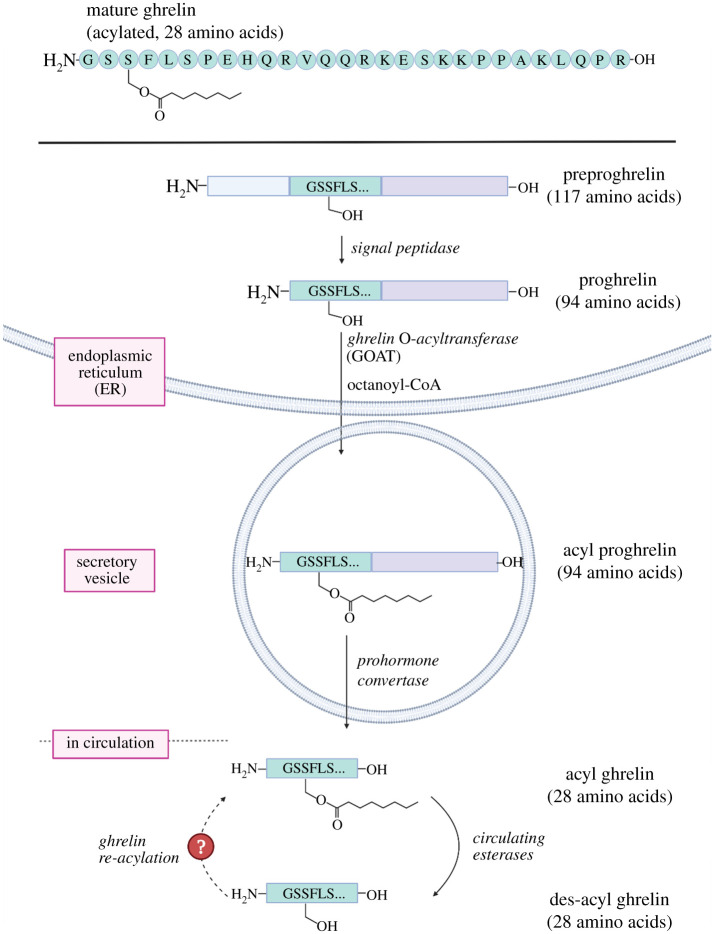
Ghrelin processing pathway involves a unique serine octanoylation modification. Several proteolytic steps and serine octanoylation by GOAT precede ghrelin secretion into the bloodstream, where it undergoes esterase-catalysed deacylation while in circulation. The dotted arrow (left) reflects the potential for local reacylation of des-acyl ghrelin by cells with plasma membrane exposed GOAT. Figure created with Biorender.com.

Like other secreted proteins, ghrelin undergoes multiple intracellular processing steps following expression before being excreted from the cell (figure 4). Ghrelin is expressed as preproghrelin, a 117-amino acid precursor containing an N-terminal secretion signal peptide. This signal moiety is cleaved by signal peptidases in the ER producing 94-amino acid proghrelin [1,85–87]. Proghrelin is uniquely *O*-octanoylated at the side chain of Ser-3 [1,3,5]. To date, ghrelin is the only protein known or predicted in the human proteome to be covalently modified with an octanoate and is the sole substrate for GOAT [2,3]. Acyl proghrelin is cleaved by prohormone convertase 1/3 (PC 1/3) after Arg-28 to produce the mature N-terminal acylated fragment known as ghrelin, which is secreted into the bloodstream [85]. A splice variant of ghrelin, coined ‘mini-ghrelin’, has also been identified [88,89]. Mini-ghrelin is expressed from a gene in which exon-2 is skipped, resulting in the expression of a preproghrelin variant that is C-terminally truncated after the first 13-amino acids of ghrelin during maturation. This splice variant is conserved in vertebrates and imitates acylated ghrelin's biological functionality in cell cultures and mice, consistent with this shorter variant undergoing the same octanoylation as full length ghrelin [88]. This functional conservation may reflect the necessity of maintaining ghrelin's multifunctional signalling role in organisms.

The Hedgehog (Hh) family of proteins has a crucial role in organismal patterning, with Sonic Hh being the most studied in mammals [90]. Hh expression produces a preproprotein containing a secretion signal, a signalling domain and a C-terminal autoprocessing domain. Upon removal of the secretion signal peptide in the ER, Hh proteins undergo autocatalytic cleavage which releases the signalling domain [91]. During this cleavage, the signalling domain is modified at its C-terminus to form an ester bond with cholesterol [92]. Contemporaneously, the Hh signal domain is modified with amide-linked palmitate at the α-amino group of the N-terminal Cys-24 by Hhat [93–95]. The dual lipidated mature form of Hh translocates through the secretory pathway and out of the cell to exert its morphogenic role. The double lipidation of Hh is posited to regulate recognition and release from cell surfaces and enhance signal transduction [96,97].

Wnt ligands encompass a family of 19 cysteine-rich proteins with regulatory functions affecting early cell development such as proliferation, specification, migration and mature cell homeostasis [98]. Wnt proteins are expressed with a secretion signal sequence, which leads to trafficking to the ER where these proteins are glycosylated and acylated. Wnt is *O*-palmitoleoylated (16:1) by PORCN at a conserved serine residue within a loop defined by two disulfide bonds [99,100]. The acyl modification of Wnt is necessary for morphogen function with the Wnt receptor Frizzled and LRP5/6 [101]. The acyl moiety is also important for Wnt binding to the trafficking protein Wntless [102–105]. Mutagenesis studies abolishing Wnt acylation demonstrate that the trafficking of Wnt throughout cells becomes impaired and the protein remains localized in the ER in the absence of *O*-palmitoleoylation [99,103,106,107]. Therefore, the modification of Wnt with the palmitoleate is essential for Wnt signalling, trafficking and secretion.

## 4. Biochemical studies of ghrelin acylation by GOAT

### 4.1. Ghrelin recognition by GOAT

The location and composition of substrate-binding sites and active site within GOAT and other protein-modifying MBOAT family members have remained undefined. However, multiple research groups have used functional studies to clearly identify the amino acids and functional groups in ghrelin that are important for recognition by GOAT. In one of the original reports of GOAT's discovery, mutagenesis of proghrelin suggested the N-terminal sequence of this ghrelin precursor contains the motif necessary for GOAT recognition [3]. A synthetic peptide consisting of the first five amino acids in proghrelin (GSSFL-[NH_2_]) is efficiently octanoylated in *in vitro* assays at levels similar to the full substrate, again consistent with this sequence acting as the GOAT recognition element [108]. Further structure-activity analyses using modified synthetic peptides fully defined the specific functional groups within ghrelin that GOAT requires for binding its peptide substrate [2,109–112].

Moving through the ghrelin N-terminal sequence, the N-terminal glycine residue is recognized by both the N-terminal amino group and the absence of the side chain at the alpha carbon [2,108,110,112]. The serine at the second position is not essential for modification by GOAT, but the size and hydrogen bonding character of the side chain at this position is read out by GOAT [2,108,110,112]. GOAT unambiguously identifies and acylates the side chain hydroxyl of Ser-3 with no acylation of the neighbouring serine if the acylation site is mutated to alanine [2,3,108,110]. When Ser-3 is replaced with threonine, as is observed in some amphibians, acylation occurs by the human and mouse isoforms at a reduced level [108,110,113,114]. GOAT naturally catalyses *O*-octanoylation of ghrelin but can also inefficiently catalyse *N*-acylation in engineered substrates containing an amine at the acylation site. Replacing Ser-3 with 2,3-diaminopropionic acid (Dap) results in an octanamide modification at a severely reduced level compared to substrates containing serine [111,112]. GOAT also tolerates stereochemical conversion at the acylation site and accepts *D*-serine as a substrate [112].

GOAT recognition of the phenylalanine at the fourth position (Phe-4) reflects a preference for large nonpolar amino acids in the binding site for this residue [2,112]. Interestingly, GOAT is severely stereoselective of Phe-4 with a complete loss of binding upon substitution with D-phenylalanine [112]. Though not as essential as the *N*-terminal ‘GSSF’ sequence, additional interaction sites further down in the proghrelin sequence may contribute to GOAT binding to a smaller degree [2,111]. Bioinformatic analysis based upon GOAT's recognition requirements concluded ghrelin to be a unique substrate for GOAT in the human proteome [2].

### 4.2. The octanoyl-CoA acyl donor: recognition by GOAT and physiological source of substrate

Since the discoveries of ghrelin and GOAT, the preference for *n*-octanoyl modification and exclusion of longer chain fatty acids has marked GOAT as unique among MBOAT family members [2,5,115]. Although ghrelin was discovered with an *O-n*-octanoyl modification of its third serine, studies have demonstrated that GOAT can use a variety of medium-chain fatty acids when the preferred eight-carbon substrate is absent and alternative fatty acids or triglycerides are provided in the cell media or animal diet [1,5,116–118]. For example, acylated forms of ghrelin ranging from two-carbons up to ten-carbons were observed by mass spectrometry in fatty acid supplemented cells expressing the mouse isoform of GOAT and ghrelin [5]. However, studies using both acylated ghrelin mimetic inhibitors and acyl donor competition experiments reveal a strong preference for the eight-carbon octanoyl chain for binding and reaction with GOAT [2,115].

**Figure 5.**
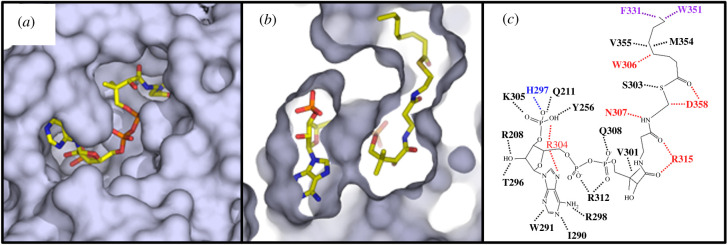
The GOAT acyl-donor binding site selects for an eight-carbon acyl chain. (*a*) The acyl-donor binding site is exposed on the cytoplasmic face of GOAT; (*b*) Cutaway view of the acyl chain of octanoyl-CoA binding into a hydrophobic pocket within the enzyme internal channel. (*c*) Alanine mutagenesis of residues contacting octanoyl-CoA leads to either reduction (purple) or complete loss (red) of enzyme acylation activity. Figure reproduced from reference [115] under the terms of the Creative Commons CC-BY licence.

Since the discovery of MBOAT-catalysed protein acylation, the mechanism by which cytoplasmic resident acyl-CoA donors are accessible for modification of luminal protein substrates was identified as a key challenge in understanding the operation of these enzymes. In the case of Hhat, recent work demonstrated that this enzyme promotes palmitoyl-CoA uptake across the endoplasmic reticulum membrane as a mode of activity that could be emulated by other MBOATs [119]. The computationally determined structure of GOAT supports another strategy for achieving transmembrane protein acylation [115]. In the structure of GOAT complexed with octanoyl-CoA, an acyl binding pocket for the octanoyl chain is present within the interior of GOAT with enzyme residues forming multiple contacts and the lipid-binding pocket capped by large aromatic residues Phe-331 and Trp-351 (figure 5) [115]. Interestingly, mutagenesis of either of these residues alters the enzyme's acyl-donor preference towards longer chain lauryl (C12) and myristoyl (C14) acyl-CoAs compared to the eight-carbon chain preferred by wild-type GOAT. In contrast with the nonpolar acyl binding site within GOAT, the phosphopantetheine and phosphoadenosine portions of the acyl-CoA donor form contacts with polar residues including an MBOAT-conserved asparagine residue (307 in GOAT) near the cytosolic interface surface of the enzyme [7,115].

When considering the octanoyl-CoA acyl donor, studies support roles for both exogenous and endogenous sources for this essential ghrelin acylation cosubstrate. Multiple studies indicate nutrient consumption acts as an exogenous source of medium-chain fatty acids (MCFAs) including octanoate [116–118]. For example, animal studies which introduced unnatural heptanoic acid into the food source produced ghrelin with acyl chains derived from the ingested diet [117]. Looking beyond dietary sources, ghrelin-producing cells treated with inhibitors of fatty acid β-oxidation exhibit decreased levels of acylated ghrelin [120]. This suggests that β-oxidation of long-chain fatty acids can serve as an endogenous source for octanoate to be used for ghrelin acylation. This same study explored the potential impact of the intestinal microbiome to produce MCFAs for use in ghrelin acylation but did not find data to support this mechanism for supplying the acyl donor for ghrelin modification.

## 5. GOAT structural studies

### 5.1. Defining the membrane topology and three-dimensional structure of GOAT

GOAT is a structurally complex polytopic integral membrane protein like its MBOAT relatives. The membrane topology of the mouse GOAT isoform was determined in 2013 by Taylor *et al*. [82] using a combination of bioinformatic analysis and selective membrane permeabilization experiments to define transmembrane domain boundaries. GOAT was determined to contain 11 transmembrane domains and a reentrant loop and photocrosslinking studies indicate the C-terminal domains of the enzyme form the substrate-binding sites [82,109]. Subsequent membrane topology studies of Hhat revealed strong conservation of the C-terminal domain structure between these two related enzymes [83,84].

**Figure 6.**
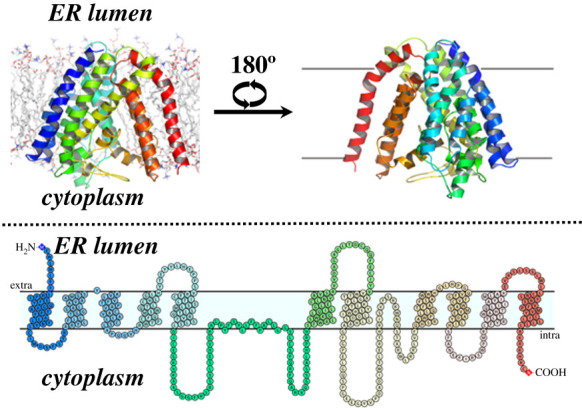
Computational model of human GOAT structure. The structure of hGOAT is shown in an ER-mimetic lipid membrane, with helices colour coded to the accompanying membrane topology diagram. Figure reproduced from reference [115] under the terms of the Creative Commons CC-BY licence.

Studies to define the three-dimensional structure of GOAT have been confounded by its integral membrane nature, leading to challenges for the purification of active enzyme. Limited success with detergent solubilization using Fos-16 led to the purification of inactive enzyme [109,111]. To circumvent these challenges, Campaña *et al*. [115] combined computational and biochemical approaches to create a structural model for human GOAT (figure 6). Initial modelling constraints were derived from coevolutionary contact analysis, which relies on amino acid co-conservation to define distance constraints that can be used for protein structure folding and prediction. Following computational folding, the GOAT structure was energy optimized in an ER-mimetic phospholipid bilayer using molecular dynamics. The GOAT structural model was consistent with the topology reported for mouse GOAT [82], with the transmembrane helices converging at the ER luminal surface to form a tent-like structure. The cytoplasmic loops domains fold up within the perimeter of transmembrane helices, resulting in very little of GOAT's structure extending past the plane of the ER membrane. Most striking, the GOAT model predicted the presence of an internal channel transiting through the enzyme from the ER to the cytoplasm. Many conserved residues contact this internal channel, and extensive alanine mutation studies confirm the functional necessity for amino acids lining this catalytic tunnel. The GOAT model also defined the acyl-donor binding site with sufficient precision to allow substrate selectivity alteration through targeted mutagenesis, as discussed above in §4.2. As the only published structure of a protein-modifying member of the MBOAT enzyme superfamily, this GOAT structure provides valuable insight into the molecular architecture underlying MBOAT-catalysed protein acylation.

### 5.2. Structural comparison of GOAT to other MBOAT family members

**Figure 7.**
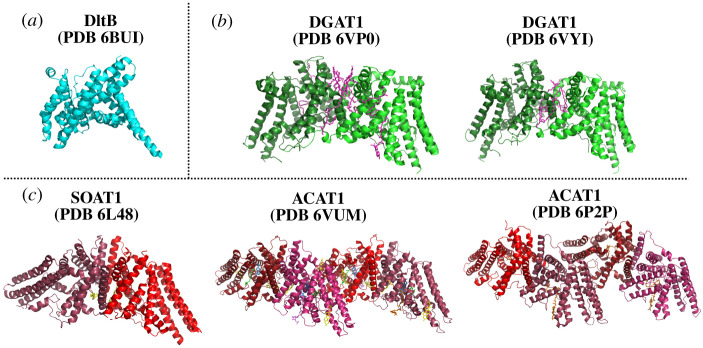
Experimentally determined structures of MBOAT family members. The structure of DltB (PDB 6BUI) was determined by X-ray crystallography, with the other MBOAT structures solved by cryoelectron microscopy (DGAT1: PDB 6VPO [121], PDB 6VYI [122]; ACAT1/SOAT1: PDB 6L48 [123], PDB 6VUM [124] and PDB 6P2P [125]). Cryo-EM structures of DGAT1 solved by two laboratories report similar dimeric structures. ACAT1/SOAT1 was solved by three different laboratories, with two groups reporting tetrameric structures and the remaining group yielding a ACAT1/SOAT1 dimer. (*a*) DltB is shown in cyan. (*b*) DGAT, with each monomer in the dimer denoted by shades of green. Lipids are shown in magenta. (*c*) ACAT/SOAT, with each monomer depicted in shades of red. O-succinylbenzoyl-N-CoenzymeA, orange; cholesterol, yellow; nevanimibe, purple and blue; coenzyme A, violet; lipids, green. Figure created with PyMol.

In parallel to the computational modelling studies of GOAT, recent progress in structural studies of several small-molecule-modifying MBOAT family members has begun to bring the molecular architecture of these enzymes into focus (figure 7). Structures of the bacterial MBOAT DltB, mammalian diacylglycerol *O*-acyltransferase 1 (DGAT1) and mammalian sterol *O*-acyltransferase1 (SOAT1/ACAT1) acyltransferases provide the opportunity to compare structural and proposed mechanistic features across these divergent members of the MBOAT superfamily. While there is significant variation in multiple aspects of enzyme oligomerization state, substrate identity, bindings sites and peripheral structural elements among these enzymes, we can begin to draw conclusions regarding conserved features across these acyltransferases.

The first published MBOAT structure described the bacterial D-alanyltransferase DltB from *Streptococcus thermophilus* in 2018 [126]. DltB performs D-alanylation of teichoic acid as part of cell wall biosynthesis. In contrast with GOAT and several other MBOATs, DltB requires a partner protein DltC which serves as an acyl carrier protein in the process of D-alanylation [83,115,119,126–129]. The crystal structure of DltB revealed 11 transmembrane helices, and both the DltB crystal structure and hGOAT model reveal a tent-like overall structure with a similar overall topology. While DltB shares low homology with hGOAT (12.3% sequence identity, 26.8% sequence similarity), structural alignment of the most conserved 100 residues in the two structures reveals a high level of similarity between these distant relatives with an RMSD of 2.23 angstroms [115]. Notably, this alignment nearly perfectly overlays the absolutely conserved histidine residues in these two enzymes inside an internal channel consistent with this residue playing a catalytic role within the acyltransferase active site.

Human DGAT1 (hDGAT1) is responsible for acylating diacylglyerol in the triglyceride biosynthesis. Following purification of DGAT1 employing solubilization by two detergents, digitonin and lauryl maltose neopentyl glycol (LMNG), the structure of DGAT1 was determined by cryo-EM [121,122]. In the cryo-EM structures, hDGAT1 is a dimer that forms a butterfly-like structure with nine transmembrane helices per monomer. In each monomer, transmembrane helices 2 thought 9 (TM2–9) structurally orient to form a ‘MBOAT fold’ enclosing a central pore [121,122]. The first 20 resolved residues on hDGAT1 are responsible for the dimer formation, with the N-termini of each monomer interacting with the MBOAT fold of the opposing monomer. hDGAT1 dimer formation is essential for enzyme activity as it is required to form the enzyme active site [121,122]. In each hDGAT1 monomer, a lipid exposed lateral gate allows access to the reaction site within the enzyme [122]. This lateral gate, currently a unique feature to hDGAT1 among MBOAT family members, is proposed to serve as an entrance for membrane-associated DAG substrates and as an exit for triacyl glyceride following the acylation reaction.

The structure of the fellow MBOAT human sterol *O*-acyltransferase (hSOAT1 / hACAT1) was also solved by cryo-EM in 2020 [123–125]. For hSOAT1, enzyme solubilization and purification were accomplished using glycol-diosgenin (GDN), digitonin and LMNG [123–125]. Similar to hDGAT1, hSOAT1 has 9 transmembrane helices and an N-terminal domain that plays an essential role in interactions between monomers. However, the structure of this N-terminal domain was not resolved in the hSOAT1 tetrameric structure. Tetramer formation is required for an enzymatic activity like the behaviour observed with hDGAT1 [123–125]. hSOAT1 contains a similar ‘MBOAT core’ transmembrane topology and an internal tunnel similar to the other MBOATS with contains the conserved histidine residue [125].

As more structures of MBOAT family members are solved, common features among these enzymes are starting to emerge. All MBOAT family members contain an internal tunnel that leads to the proposed active site where the conserved functionally essential histidine resides. In some members such as GOAT and DltB, this channel completely transits the enzyme structure to form a transmembrane channel. Other MBOAT family members such as DGAT1 and SOAT1 contain a lateral tunnel or lateral gate to the active site from the enzyme interface with the surrounding membrane bilayer. These lateral chambers meet up with the transmembrane tunnel near the conserved histidine residue. We look forward to further comparisons between MBOAT structures as more become available, with a particular interest in comparing the active sites and catalytic strategies employed by the protein-modifying members of this intriguing enzyme superfamily.

### 5.3. Structural insights into the mechanism for GOAT-catalysed ghrelin acylation

**Figure 8.**
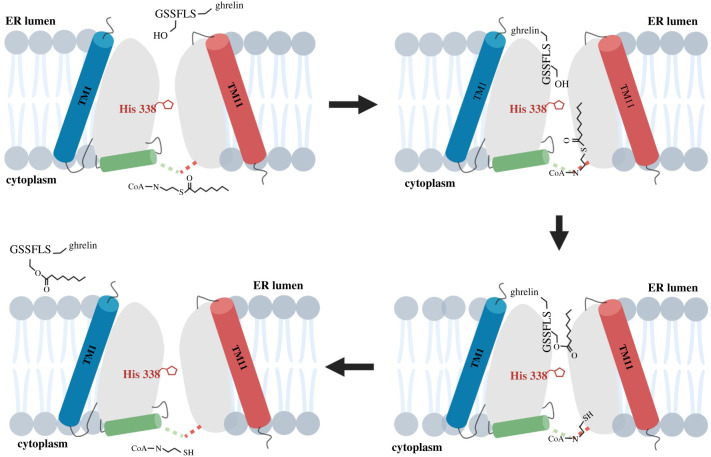
Proposed reaction cycle for transmembrane ghrelin octanoylation by GOAT. The two substrates for GOAT, ghrelin (GSSFL-ghrelin) and octanoyl-CoA, enter the enzyme internal channel from the ER lumen and cytoplasm, respectively. Acyl transfer of the octanoyl chain to the ghrelin serine hydroxyl is catalysed by interaction with His-338, followed by octanoylated ghrelin dissociation to the ER lumen and coenzyme A release back to the cytoplasm. Figure created with BioRender.com.

The catalytic mechanism for ghrelin acylation by GOAT has not been conclusively characterized, but the combination of functional studies with recent structural modelling has led to an intriguing model for this topologically challenging protein modification (figure 8). In their original identification as an enzyme superfamily, MBOAT members were shown to contain two highly conserved residues, an asparagine or aspartate approximately 40 amino acids distant from an invariant histidine in the enzyme C-terminal [7]. These residues were originally thought to play roles in catalysis based primarily on their high conservation among MBOAT members, with the loss of GOAT acylation activity upon mutation of these residues (Asn-307 and His-338 in GOAT) supporting this proposal [3,5,7]. However, topological studies of mGOAT demonstrated that these two residues are separated by the ER membrane and are unlikely to reside near each other in an active site within GOAT [82]. Interpreted in light of available biochemical data, the computational structural model of hGOAT suggests distinct essential roles for these two residues in GOAT substrate binding and catalysis. Previous proposals for His-338 serving as a general base are well supported by both functional and structural data. As noted above, multiple studies have shown mutation of His-338 leads to a complete loss of GOAT acylation activity [3,5,108,111]. In the computationally derived structure, His-338 is proposed to contact the luminal pore within the core of hGOAT positioned similarly to the analogous histidine (His-336) in the crystal structure of the bacterial MBOAT alanyl transferase DltB [115,126]. In GOAT, this positioning could allow His-338 to contact the acylation site hydroxyl of ghrelin as the peptide enters the enzyme from the ER lumen. Near the cytoplasmic face of the enzyme, the other conserved residue, Asn-307_,_ interacts with the octanoyl-CoA acyl donor as it enters the enzyme from the cytosol. This model explains how the topologically separated residues His-338 and Asn-307 play complementary but distinct functionally essential roles in ghrelin acylation by GOAT.

With the octanoyl chain bound within the core of hGOAT in the acyl binding pocket, the octanoyl-CoA thioester carbonyl can be potentially arranged for attack by the activated ghrelin serine β-hydroxy nucleophile leading to direct transfer of octanoate to ghrelin. Alternatively, GOAT could proceed through a two-step ping-pong mechanism involving an acyl enzyme intermediate as seen with DHHC acyltransferases [130,131]. This latter mechanism requires one (or more) nucleophilic residues within the vicinity of the lipid-binding site. Mutagenesis studies of residues lining the GOAT internal channel show that many of these residues are required for enzyme function, supporting further mechanistic studies to formally elucidate their role(s) in GOAT catalysis [115]. Whether proceeding by direct transfer or through an octanoyl-enzyme intermediate, the acylation reaction concludes with the acylated ghrelin product departing the enzyme channel and returning to the ER lumen and CoA being released back to the cytosol.

Comparison of the computational GOAT structural model to recent structures of DGAT1 and SOAT1 indicates that some aspects of the proposed mechanism for GOAT-catalysed ghrelin acylation are likely conserved across MBOAT family members. DGAT1 catalyses the synthesis of triacylglycerols using diacylglycerol and fatty acyl-CoAs as substrates [127], and SOAT1 uses long-chain acyl-CoAs to catalyse the transfer of lipids to form cholesterol esters [128]. Notably, neither of these small-molecule-modifying MBOATs appear to use the transmembrane channel observed in GOAT and DltB suggesting that the topological constraints on acylation differ across the MBOAT superfamily. Although the diacylglycerol and cholesterol acyl acceptors of DGAT1 and SOAT1 differ from GOAT's protein substrate ghrelin, the proposed mechanism of catalysis in all three enzymes involves the invariant histidine acting as a general base to generate an activated hydroxyl nucleophile on the acyl acceptor substrate—the *sn*-3 free hydroxyl of diacylglycerol in DGAT1 and the 3β-OH of cholesterol in SOAT1 [121,124]. In both of these other enzymes, the conserved asparagine residue (Asn-378 in DGATI, Asn-421 in SOATI) lie in the vicinity of the thioester bond of the acyl-CoA substrate and may act to stabilize the acyl donor for attack by the activated nucleophile. Continuing modelling and structural studies of GOAT and other MBOAT family members remain an active endeavour towards defining the mechanism of protein and small-molecule acylation by these enzymes.

## 6. Ghrelin modification following secretion

### 6.1. Ghrelin deacylation by circulating esterases

**Figure 9.**
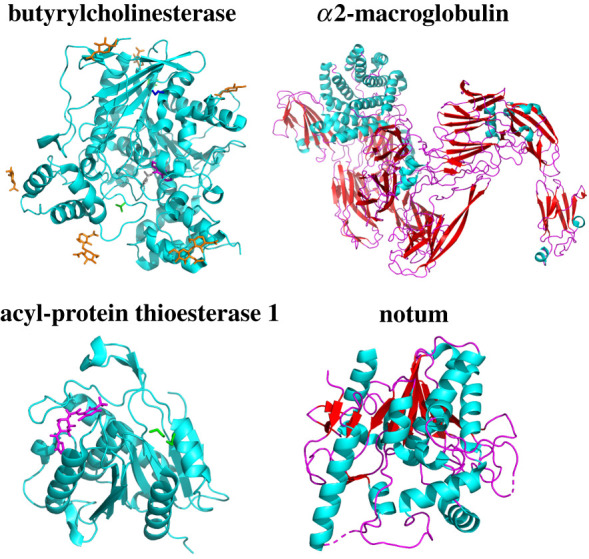
Esterases in circulation with demonstrated ghrelin deacylation activity. Each of these esterases has been shown to hydrolyse the octanoyl serine within GOAT. Butyrlcholinesterase (PDB 4BDS); *α*2-macroglobulin (PDB 5A42); APT1 (PDB 5SYM); Notum (PDB 4WBH). Figure created with Pymol.

**Figure 10.**
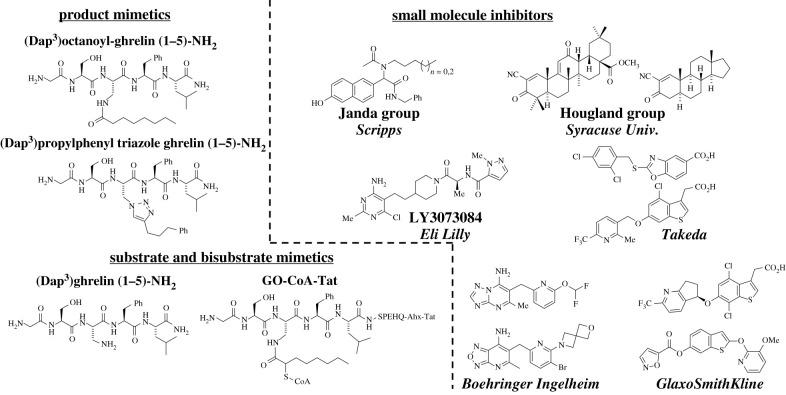
Representative classes of GOAT inhibitors. The current range of reported GOAT inhibitors can be divided into three main classes: product-mimetic peptide inhibitors, substrate and bisubstrate-mimetic peptide inhibitors, and small-molecule ‘drug-like’ inhibitors.

Following acylation by GOAT and secretion, acyl ghrelin circulates in the bloodstream to activate endocrine signalling at a number of sites within the body. While ghrelin remains in the bloodstream, it also undergoes deacylation by circulating esterases [132–137]. While a specific ghrelin esterase has not been identified, several candidates have been characterized and are proposed to play roles in ghrelin signalling (figures 9 and 10). One of the most compelling candidates is butyrylcholinesterase (BChE), which was annotated as a ghrelin esterase upon finding that mice exhibited a BChE-dose-dependent decrease in ghrelin levels [132,138–140]. Subsequent studies using purified BChE support that this enzyme exhibits sufficient ghrelin ester hydrolysis activity to serve as the ghrelin esterase within the bloodstream [132,141]. *α*2-macroglobulin was identified as another ghrelin esterase in rat serum using a ghrelin mimetic functionalized with a privileged serine electrophile warhead [133]. This suggests that *α*2-macroglobulin has a recognition site for ghrelin binding. In *in vitro* studies, *α*2 -macroglobulin catalyses deacylation of ghrelin with a K_m_ of 24 ± 3 µM and a k_cat_ of 2.3 × 10^−2^ ± 0.1 × 10^−2^ min^−1­^ which was argued to provide sufficient activity for physiological relevance [142]. Acyl-protein thioesterase 1 (APT1)/lysophospholipase I was identified as a third ghrelin esterase following its purification from fetal bovine serum (FBS) and was shown to deacylate ghrelin *in vitro* [135]. In both cell- and animal-based studies, APT1 release and serum ghrelin deacylase activity were increased following treatment with lipopolysaccharide (LPS). This suggests APT1 can play a role in regulating ghrelin deacylation under conditions of infection and septic shock, with further studies needed to explore these proposed mechanisms for linking infection response and ghrelin signalling. Most recently, the Wnt deacylase Notum has been reported to also catalyse hydrolysis of the serine octanoyl ester within ghrelin [137,143]. As Wnt signalling is also involved in energy regulation through fat and glucose metabolism, Notum has the potential to play a significant role in multiple processes influenced by distinct secreted acylated proteins [137,143,144]. Given the central role of ghrelin deacylation in controlling ghrelin signalling, identifying and characterizing ghrelin esterase is essential for building a global understanding of ghrelin's impact throughout the body.

### 6.2. Ghrelin re-acylation: potential mechanism for signalling by des-acyl ghrelin

The accepted view of ghrelin signalling activity involves secretion into circulation followed by either signalling at distal sites within the body though the GHS-R1a receptor or ghrelin deactivation through deacylation. However, recent studies have provided evidence to support another branch in ghrelin signalling through ghrelin re-acylation by locally expressed GOAT at the signalling site (figure 4) [67,79]. In 2017, immunogold electron microscopy of rat blood marrow adipocytes detected GOAT in the membrane of lipid trafficking vesicles and the plasma membrane [67]. This suggests GOAT can regulate des-acylated and acylated ghrelin beyond the secretion pathway following ghrelin translation [82]. This study reported bone marrow adipocytes showed adipogenesis activity when treated with both ghrelin and des-acyl ghrelin, with this adipogenesis activity abolished in GHS-R1a knockout mice [67]. Local re-acylation of des-acyl ghrelin would require localization of GOAT at the cell surface, a notion that challenges the current understanding of the cellular localization of the enzyme responsible for the acylation of ghrelin [49,82].

In a second study, Murtuza *et al*. [79] identified GOAT activity at the plasma membrane of hippocampal neurons. Incubating hippocampal slice cultures with fluorescently labelled acyl and des-acyl ghrelin peptides lead to membrane-bound fluorescence in both cases attributed to ghrelin binding to the GHS-R1a receptor. Incubation with a GOAT inhibitor or treatment of fixed cells led to the loss of fluorescent cell labelling with the des-acyl ghrelin probe. This suggests that enzymatically active GOAT is present in the plasma membrane of hippocampal neurons [79].

## 7. Pharmacological control of ghrelin signalling: GOAT inhibitor development

Ghrelin initially gained recognition as the appetite-stimulating (orexigenic) hormone that could peripherally regulate hunger signals, thereby making the ghrelin-signalling pathway an attractive means to potentially impact the current obesity epidemic. Since ghrelin's discovery in 1999, it has been found to be involved in an increasing variety of physiological and metabolic processes [1,9,26,27,71]. The exclusivity of GOAT's catalysis of ghrelin octanoylation and the requirement for ghrelin acylation to become biologically active highlight GOAT as an appealing therapeutic target for controlling ghrelin signalling. Although GOAT was identified as the enzyme responsible for ghrelin's activation since 2008, the lack of potent GOAT inhibitors until recently has impeded the exploration and exploitation of GOAT as a therapeutic avenue for modulating ghrelin-related pathologies [145,146]. Recent progress in GOAT inhibitor development will support ongoing studies to leverage ghrelin and GOAT for treating diseases such as diabetes, obesity and addictive behaviour.

### 7.1. Ghrelin mimetic inhibitors

Biochemical characterization studies of GOAT were critical in identifying the first inhibitors of GOAT acylation of ghrelin. These inhibitors were synthetic peptidomimetics with features compared to the N-terminal sequence of proghrelin, GSSF-[NH_2_], which was identified as the recognition motif [108]. GOAT octanoylation of proghrelin was decreased when the enzyme was incubated with an octanoylated form of a N-terminal pentapeptide derived from ghrelin, GSS(C8:0)FL-[NH_2_], with an IC_50_ of 45 µM. Substitution at the acylation site serine of the truncated peptide with a diaminopropanoic acid (Dap) yields greater inhibition of GOAT at sub- to low-micromolar concentrations when using the hydrolytically stable octanoylated form, GSDap(C8:0)FL-[NH_2_] [108]. Permutation of the amide-linked acyl chain of the pentapeptide inhibitor demonstrated GOAT has a marked preference for moderate length acyl chains. Inhibitors bearing an eight-carbon chain were most potent for inhibiting GOAT, with both increasing and decreasing acyl chain lengths reducing inhibitor activity [2]. A series of novel product-mimetic inhibitors incorporating a 1,2,3-triazole linkage to hydrophobic alkylphenyl groups at the acylation site also acted as potent GOAT inhibitors [147]. These triazole-containing inhibitors inhibited GOAT at sub-micromolar concentrations, indicating GOAT's tolerance for replacement of ester or amide bonds at the ghrelin acylation site with a biostable triazole linkage.

While early peptide-based ghrelin mimetics exhibited pronounced *in vitro* inhibition of GOAT, there remained concerns whether potency can be maintained in a cellular or organismal context due to concerns with peptide cell permeability and biostability. A bisubstrate-mimetic analogue, GO-CoA-Tat, was developed in which the first 10-amino acids of ghrelin are linked with octanoyl-CoA through an amide bond and a cationic TAT sequence is appended to the end of ghrelin sequence to improve cell permeability [109]. GO-CoA-Tat selectively inhibits ghrelin acylation in *in vitro* and cell assays without activating GHS-R1a receptor at sub- to low-micromolar concentrations [109]. Glucose tolerance and weight management were observed in mice maintained on a high-fat diet as a result of treatment with Go-CoA-Tat [109], and subsequent studies further demonstrated the ability of GO-CoA-Tat to modulate ghrelin levels to impact associated physiological processes and behaviours [148,149]. While not a candidate for further pharmacological development due to its large size and peptidic character, GO-CoA-Tat provided a proof-of-principle demonstration for the therapeutic role inhibition of acyl ghrelin production can provide.

In addition to the product and bisubstrate-mimetic inhibitors, we recently reported that ghrelin mimetic peptides incorporating an unmodified Dap residue in place of the acylation site serine act as potent GOAT inhibitors [112]. These substrate-mimetic inhibitors provided a platform for scanning the ghrelin N-terminal sequence for binding recognition elements using backbone amide methylation, unnatural amino acid substitutions and D-amino acids. These peptides exhibited high potency against GOAT, with IC_50_ values as low as 62 nM [112]. The potent binding of Dap-containing short peptides lacking an acyl modification has the potential to support the development of inhibitors targeting the ghrelin-signalling pathway with high affinity for GOAT without binding to the GHS-R1a ghrelin receptor.

### 7.2. Small-molecule inhibitors

Many early GOAT inhibitors were peptide-based substrate or product mimetics that, except for GO-CoA-Tat, either lacked potency in cell-based assays or were not tested in these assays. Though GO-CoA-Tat exhibits promising results, as noted above biostability and potential for further inhibitor optimization remain a concern with peptidomimetics. Consequently, small-molecule GOAT inhibitors have been sought as more likely candidates for therapeutic development and deployment. Recent progress in this area brings hope that GOAT's value as a treatment avenue targeting ghrelin-dependent health conditions may be validated in the near future.

The first reported small-molecule non-peptide-based GOAT inhibitors were synthesized and identified in 2011 from a library screen of compounds based on naphthalene analogues containing varying alkyl chains [150]. These compounds inhibit GOAT at low-micromolar concentrations in a cat-ELCCA assay that functionally mimics ELISA assays [151]. The inhibitors are hypothesized to antagonize catalysis by blocking octanoyl-CoA binding due to their lipophilic nature, although this was not verified by competition experiments. No inhibition data for these inhibitors against GOAT have been reported in cell- or animal-based studies.

Our research group screened a diverse small-molecule library and identified a set of synthetic triterpenoids that inhibit GOAT [152]. These molecules, derived from 2-cyano-2,12-dioxoleana-1,9(11)-dien-28-oic acid (CDDO), reversibly inhibit GOAT with the most potent compound in this class exhibiting an IC_50_ = 8.4 µM. Structure-function analysis demonstrated the presence of an α-cyano-enone Michael acceptor is essential for inhibition and implicates the involvement of a nucleophilic residue, possibly cysteine, in the inhibition mechanism. Interestingly, inhibition was observed in the human isoform of GOAT but not in the highly similar mouse isoform (79% identical, 92% similar). This contrast in inhibition profile suggests these inhibitors target a non-conserved residue and binding site in hGOAT, which would argue against direct engagement with the enzyme active site. Interestingly, one of the original CDDO derivative compounds found to inhibit GOAT, bardoxolone methyl, has been assessed as a therapeutic for inflammation and oxidative stress acting through the Nrf2-KEAP pathway. Clinical studies with bardoxolone methyl reported side-effects such as weight loss, reduction of diet-induced insulin resistance and improvements to glucose tolerance which are physiological effects that would be associated with alterations in the ghrelin-signalling pathway [153–156]. While the cross-reactivity of these compounds with other cellular targets limits their usefulness as specific GOAT inhibitors, it also raises the possibility that other current pharmaceuticals may be impacting ghrelin-dependent physiological pathways through off-target inhibition of GOAT acylation activity.

Potent small-molecule GOAT inhibitors developed by pharmaceutical companies have recently begun to be reported in the scientific and patent literature. A set of compounds containing an aminopyrimidine scaffold which antagonize GOAT acylation have been reported to show inhibition activity in ELISA-based assays [157–161]. The compounds were further shown to be competitive with octanoyl-CoA but not ghrelin. Compound GLWL-01 (formerly LY3073084) was developed following optimization studies to increase *in vivo* biostability and was determined to have an IC_50_ of 69 nM while exhibiting good PK/PD properties [162]. This compound is currently undergoing clinical trials as a therapeutic for several disorders [163–165]. Using a homogeneous time-resolved fluorescence ELISA, Yoneyama-Hirozane *et al*. [166] identified a lead GOAT inhibitor-containing heterocyclic aromatic rings. Both the initial hit compound and an optimized derivative acted as nanomolar GOAT inhibitors. The compounds were effective at inhibiting both the human and mouse GOAT isoforms and similarly, exhibited competitive inhibition with octanoyl-CoA but not des-acyl ghrelin. Mice orally dosed with the optimized GOAT inhibitor produced lower levels of serum ghrelin, and further *in vivo* studies were suggested. Wang reported another set of compounds as GOAT inhibitors based on a functionalized benzothiophene scaffold [167]. Inhibition with these compounds was observed in the nanomolar range and, when containing an ether linked pyridine decorated with methoxy and fluorine, greater than 90% inhibition was observed at 10 µM [168–170]. Godbout *et al*. [171,172] disclosed the most potent and active compounds identified as GOAT inhibitors to date, which contain an 7-amino[1,2,5]oxadiazolo[3,4,-b]pyridine scaffold and display picomolar inhibition.

## 8. Current challenges and future opportunities

As studies of ghrelin and its associated signalling pathway enter their third decade, the unique aspects of this peptide hormone and its impact across multiple physiological processes continue to motivate investigations. These studies span across the scientific spectrum from fundamental protein biochemistry to organismal metabolism and neuroendocrinology. At the molecular level, the most pressing challenges are the experimental determination of the structures of GOAT and the ghrelin•GHS-R1a signalling complex. Recent structures of the ghrelin receptor suggest the potential for rapid progress on this front, but the purification of enzymatically active GOAT remains a significant barrier to GOAT structural studies. The computational model of GOAT answered several questions regarding the enzyme architecture and revealed the transmembrane catalytic channel. Future studies by crystallography or cryo-EM will provide valuable insights into the substrate-binding sites and enzyme active site. We must also determine if GOAT itself is subject to regulation by posttranslational modifications through either direct impact on enzyme activity or effects on GOAT localization and trafficking across cellular membranes.

Beyond expanding our understanding of the catalytic strategies employed by protein-modifying MBOAT family members, defining GOAT's structure will also inform inhibitor design and optimization. Potent and specific GOAT inhibitors are essential molecular tools for probing ghrelin's impact within the body as we move towards a complete model of ghrelin signalling at the organismal level. This model must address the biological role of des-acyl ghrelin, ghrelin reacylation at signalling target sites, the influence of ghrelin on behaviour and mental health, and all other aspects of ghrelin signalling within the body.

Recognized at its discovery for its unique role in physiology and its potential as a therapeutic target, recent advances in the ghrelin/GOAT/GHS-R1a field have positioned us well to leverage opportunities to understand and harness ghrelin signalling. The creation of multiple potent GOAT inhibitors now enables the validation of ghrelin signalling as a treatment avenue for multiple metabolic disorders such as diabetes and obesity. At the molecular level, we can also now explore why ghrelin bears its unique octanoyl serine modification—perhaps as a mechanism for decoupling ghrelin signalling from the metabolic state of the ghrelin-producing cell by forcing the use of an exogenously derived acyl donor? The strong conservation of ghrelin and GOAT across the vertebrates also suggests an essential role for signalling, perhaps as a ‘survival hormone’ enhancing the ability of an organism to respond to multiple environmental stressors such as starvation over both acute and chronic time scales.

Moving beyond metabolism and energy homeostasis, ghrelin can also provide a new avenue for understanding and treating addiction, stress and anxiety disorders, and other neurological conditions impacted by ghrelin signalling. The growing complement of molecular tools for modulating and blocking ghrelin signalling provides the ability to uncover and understand the involvement of this acylated hormone in a growing range of processes at the cellular and organismal level. Continuing efforts across the biochemical, biological and medical ghrelin research communities are essential for realizing these opportunities for defining and exploiting ghrelin signalling for therapeutic effect. The acceleration of progress across these fields in the last 5 years suggests we are entering an exciting new era in our understanding of ghrelin and its biological impact.

## Data accessibility

This article has no additional data.
